# Lectures on the Nature and Treatment of Deformities

**Published:** 1846-04

**Authors:** 


### Lectures on the Nature and Treatment of Deformities. By *R. W. Tamplin*, F.R.C.S.E., Surgeon to the Royal Orthopædic Hospital. Small 8vo. pp. 267. London, 1846. Longman and Co.

It is now nearly 30 years ago since Delpech introduced the present mode of treating deformities of the feet, by section of the contracted tendons and the subsequent application of regulated extension. The experience of surgeons, since the appearance of his very able work entitled Orthomorphie, in 1828, has amply confirmed the complete truth of the principles which he then laid down, and the soundness of the practice which he based upon them. These principles are comprised in the following rules:

"1st. 'A tendon to be divided must not be exposed; and its division should be made by turning the instrument on one side, so that the line of the incision may not be parallel to the division of the skin; without this precaution risk of exfoliation of the tendon is incurred.'

"2nd. 'Immediately after division of the tendon, the divided ends should be brought into contact with each other, and kept in this position by a suitable apparatus during the entire period necessary for their union.'

"3rd. 'Inasmuch as it can only take place by the intervention of an intermediate fibrous substance, this substance, before it has become firm, can, and should be, extended gradually and carefully, until it has assumed a degree of length equal to the shortened muscle.'

"4th. 'When this degree of extension has been effected, the parts should always be fixed in the position, and kept so until the new substance has acquired its requisite degree of consolidation.'" P. 4.

These simple positions may be said to embody the entire doctrine of the treatment of deformities, and, as our author remarks, "have only to be followed out carefully to insure success." Stromeyer, while he admits the general truth of M. Delpech's views, has objected to the third of his positions; viz. that the elongation of the contracted muscle is attributable altogether to the intermediate fibrous substance, deposited between the cut ends of the tendon. He maintains that this elongation is rather effected at the cost of the contractility of the affected muscle; the division of its tendon acting not only on the mechanical, but also on the vital, properties of the muscular fibres. Mr. Tamplin does not, it would seem, entirely agree with the Hanoverian surgeon in this remark. He has repeatedly found the new connecting medium, after section of the tendo-Achilles, to be full two inches in length within three or four weeks after the operation. In cases also of fractured patella, the uniting substance is sometimes, when the limb is used too soon after the accident, three inches at least in length. If such, however, be the case, the patient will inevitably be more or less crippled in the extremity.

"As a general rule, the cicatrix, after the cure is effected, measures but a few lines, oftentimes only a line, in the thickness, provided proper care has been taken during the treatment, which arises from the circumstance, that every new substance, after it has fulfilled the purpose for which it was generated, viz. the restorative process adopted by nature to remedy any wound or injury, loses its vascularity, except so far as is necessary for its individual vitality, contracts upon itself, and draws with it, if unrestrained, the part with which it is connected; as in the painful distortions so frequently witnessed in burns on the neck and face. In the foot I have found the permanent cicatrix full two inches in length (after the operation of talipes equinus); and a worse distortion produced, viz. the talipes calcaneus valgus, where the foot was placed in position immediately after the operation, and kept for a long time in the flexed position: this position was not, however, maintained, except during the exercise of volition. The elongation in congenital cases, and also in non-congenital cases, of young subjects, is undoubtedly effected eventually at the cost of the contractility of the muscle, but not primarily: this is a secondary result, for no new uniting medium can by possibility possess the power of drawing down the muscle, any more than the newly formed granulations following a burn can do; the cicatrix, as it contracts, certainly does so, but after the foot is brought into position; and hence the linear cicatrix. The force of the contraction of a cicatrix is sufficiently evident in the instances of burns alluded to, not only to draw down and overcome one muscle, but even a set of muscles—as the extensors of the head and neck, or of the arm, and other parts." P. 9.

With respect to the *cause* or causes of Congenital Deformities, it must be confessed, that we really know very little or nothing about the matter. As to the supposed influence of the mother's mind upon her offspring *in utero*, the conjecture scarcely deserves notice; for there is not a shadow of argument that can be adduced in its favour. Our author leans to the opinion that they are generally the result of malposition of the limbs of the unborn fœtus, in consequence, it may be, of undue or irregular contraction of the uterine parietes upon its contents; and he alludes to the fact—as in some degree favourable to this idea—that such a deformity as that of pure *talipes equinus*—whose origin could certainly not be accounted for in this manner—is never of congenital occurrence. It is generally admitted that by far the most frequent operating causes after birth are intestinal irritation and dentition: the latter may act indirectly, by primarily deranging the state of the *primæ viæ*.* Now, if this be the case, why may we not apply the same mode of reasoning to explain the occurrence of some cases, at least, of congenital deformities? Is there not good reason to believe that the meconium of the fœtus is often more or less vitiated; and, if so, why should not this source of irritation act, before as well as after birth, by inducing irregular and spasmodic contraction of certain muscles? But it is unnecessary to say more upon this subject; as we have only guesses, not facts, to offer. We shall therefore proceed to examine the practical portions of the present volume. This need not be done at any great length; as in our review of Dr. Little's work on Club-foot, a few years ago,† we gave an elaborate analysis of its contents, and were then enabled, through the kindness of the author, to illustrate the different species of this deformity with excellent woodcuts. In the present article, we shall devote most of our space to the consideration of other deformities, then unnoticed. It may therefore be considered as in some degree supplementary to the former one; so that the reader will have, in the two together, a tolerably ample summary of the entire subject of Orthopædy.

*Talipes equinus* or *horse-foot*—"so called from its anatomy corresponding to the natural anatomical relation of the foot of the horse." The patient in walking rests upon the heads of the metatarsal bones; the os calcis is forcibly drawn up; the astragalus is pushed forwards and downwards, and frequently projects on the dorsum of the foot; and the phalanges of the toes are extended, and appear set on at right angles to the metatarsal bones. The reader will have a perfect idea of the position of the member, if he calls to mind the forced and unnatural attitude of the opera-dancer, when she rests upon one foot in her pirouettes and other *merveilles de danse*. This deformity is almost always post-natal: Mr. Tamplin has never met with a congenital case of simple talipes equinus. The usual causes are "the irritation of teething, worms, any derangement of the nervous system, wounds in the calf, rheumatism, scrofulous disease in the ankle-joint, or in the substance or tendon of the gastrocnemius. Not unfrequently, however, this deformity arises spontaneously, the patient experiencing no pain or inconvenience beyond the inability to bend the foot or ankle-joint in the act of walking and retaining at the same time power over all the muscles." The affected limb is generally smaller than the sound one; its temperature too is sometimes lower, especially when there is paralysis of any of the muscles.‡ Whenever this complication exists, we must never promise too much from any operation; as the palsied muscles seldom or never recover their power.

---

* In reference to the influence of Dentition in the production of Club-foot, our author observes that the deformity "is often combined with contraction and partial paralysis of the *upper* corresponding extremity; occasionally, however, it is unaccompanied by any other deviation. Frequently, in such cases, there will be found paralysis of the anterior tibial muscle; so that the contraction would appear in these instances to arise from the balance of power being destroyed, and the gastrocnemius being thereby allowed to contract upon itself."

† Medico-Chirurgical Review for April, 1840.

‡ An important practical instruction is deducible from this circumstance.

When, by the division of the tendo-Achilles, the flexor movement of the ankle-joint is restored, " you will occasionally find, as the foot becomes flexed, that it will deviate either to one or the other side; most frequently it will *evert,* forming a sort of talipes valgus; and, upon examining the tendons, the peronei will be found contracted. If, however, it should *invert,* the posterior tibial will be the most probable cause. In either case division of the tendon must be resorted to, or the patient will be thrown either on the internal or external side of the foot, according to the state of the respective muscles. You will not be enabled at all times to discover this tendency previous to the division of the tendo-Achilles. Occasionally, however, you will find these tendons, viz. of the peroneii and the posterior tibial, tense in a simple talipes equinus, upon a forcible attempt to flex the foot. If this tension continues after keeping up the forced extension for some minutes, you may be nearly sure that the tendon is contracted; and I would advise division at the same time that the Achilles tendon is divided."

*Talipes varus.*—" The muscles, which are contracted in this deformity, and are the immediate cause of the malposition, are, 1st, the gastrocnemius, 2d, the adductors direct, viz. the anterior and posterior tibial, the extensor and flexor of the great toe, as well as the extensor and flexor communis indirectly. The peronei are elongated, or rather kept upon the stretch. The contraction of the ligaments does not apply to infants, but becomes so from position. This deformity is both congenital and non-congenital; but in a large proportion it is the most common form of congenital deformity. In the congenital, you will find no paralysis; in the non-congenital, paralysis of one or more muscles will very generally be found; and this applies to almost every non-congenital deformity. I have never seen a congenital paralytic affection of any of the muscles in any deformity, nor do I believe that it exists. In the infant you will find the size of the affected limb correspond with that of the well-formed and perfectly proportioned one; but of course, as the child grows, the foot remaining in its false position, and the play of the muscles in that foot being most limited, the muscles, as a natural consequence, become developed but very slowly; and as the child continues to grow, the limb presents the appearance of an atrophied extremity, the natural outline of well-developed muscles being entirely absent; and yet all this arises entirely from want of use, as no paralysis exists, nor any loss of the voluntary power. The temperature of the limb is natural, but the foot does not resist the effect of cold so well as the naturally-placed foot; but this, I imagine, is entirely owing to its passive condition, and not from any diminished nervous power, or loss of its proper tone, as it is not merely the motion of the ankle-joint that is limited; the muscles which move it are of necessity scarcely, if at all, able to perform their functions." P. 46.

---

" The temperature of the entire limb during the treatment is occasionally much reduced, so that it becomes very necessary to pay attention to this, as, by neglecting the temperature, comparatively slight pressure will often produce a slough, and then a wound tedious and troublesome to heal, and during the open state of which it will be necessary to suspend the treatment. * * * * * In cases," continues our author, " where I have had to encounter these sloughs, I have found the best method of treatment to be, to support the foot entirely by means of strapping: you thus prevent the weakened capillaries from becoming over distended; and if the vitality is not completely gone, the part will recover itself. And even should the vitality be destroyed, the threatened slough will frequently dry up, and terminate without any open wound." P. 40.

In a large proportion of cases of *varus*, the deformity exists in both feet. From their incapacity of regular and due exercise, the muscles of the affected leg always become considerably, and oftentimes exceedingly, atrophied. The ligaments of the knee-joint, also, are frequently so much relaxed, that the tibia may be partially rotated upon the condyles of the femur. Not unfrequently, too, the vertebral column becomes more or less irregular in cases of single varus, in consequence of the great leaning over of the body to the affected side.

Mr. Tamplin has never seen a decided or permanent cure of *varus*, even when the deviation has been slight, by the use of any mechanical contrivance applied to the foot or leg, without section of the contracted tendons. He is an advocate for the early performance of this operation in congenital cases. "I have frequently," says he, "performed it on infants of not more than five weeks of age, and should always recommend it to be done at this early age when opportunity offers."

In every confirmed case of Talipes varus it will be necessary, according to Mr. Tamplin's experience, to divide the tendons of the *gastrocnemius*, *tibialis anticus* and *t. posticus* muscles; and the plantar fascia also, if this be found decidedly contracted. The tendon of the posterior tibial muscle should be first divided—as the gastrocnemius serves to keep the foot steady. As this operation is far from being easy when the leg is fat, we shall give our author's description of it.

"The method," says he, "that I have adopted is the following:—let the child be laid horizontally on its back; and let the leg to be operated upon be everted so that you have the inner side facing directly upwards; you thus have the tendon directly before you; you will then feel for the tendon, which in a thin child can easily be felt to rise under the finger upon any attempt to abduct the foot. In a fat child, however, you will not be able to do so, for you must recollect it is the edge of the tendon which presents itself, and not the flat surface. The guide I then find successful is the internal edge of the tibia (which can at all times be felt with more or less pressure), and the tendon, as you are aware, lies directly behind it, and close to it. Having, then, the foot and leg firmly held by an assistant, you place the thumb of the left hand on the edge of the tibia, or close to it; you then, with a small scalpel, puncture the fascia by passing it perpendicularly down, and with care, or you pass the instrument too far, and puncture artery. But if you do it slowly and cautiously, you will be enabled by a sort of grating sensation to feel the fascia, in which you make a small slit; you then withdraw the scalpel, and introduce the blunt-pointed knife in a perpendicular direction also, or you will get on the tibia on one side, behind the tendon on the other, from its close approximation to the bone. As soon as you have got the knife before the musle, or what you imagine to be so, depress the handle, and satisfy yourself, by the resistance, that it is so: turn the sharp edge to the muscle, and divide it, directing the assistant at the same time to forcibly abduct the foot. You are aware that the artery lies very near to the posterior tibial and flexor digitorum muscle, and if you do not exercise great caution and judgment as to the distance, you may divide that also, which it is prudent to avoid: although at present we have seen no ill effects from complete division of the artery, yet we are not to imagine such will be always the case. You may form some idea of the proper distance by letting the knife touch the edge of the tibia first, then pass it onwards about a quarter of an inch, and divide the muscle. You must recollect that in infants muscular fibre exists almost down to the internal malleolus, so that the division will not give so sudden a sensation as of the division of tendon alone; but in all cases, I am confident, if the muscle is divided, the sensation must be communicated to the hand of the assistant. After having divided this muscle, place a piece of lint on the point of puncture, and keep the finger on it, and then proceed to divide the anterior tibial." P. 56.

The after-treatment of the foot requires most assiduous attention; and, unless the member be kept night and day everted or adducted by an appropriate instrument, there is always a strong tendency to a relapse, in consequence of the *peroneii* muscles having become weakened by their long and forced extension.

The time required for treating a bad case of Talipes varus will be found to vary, according to circumstances, from two to ten months.

*Talipes Valgus,* flat or splay foot, is the very reverse of *T. varus.* The post-natal species of this deformity generally occurs in weakly persons, who are a great deal upon their feet, more especially when they are obliged to carry heavy burdens at the same time. The muscles, that usually require to be divided—for Mr. T. has never seen a perfect or permanent cure effected by the mere use of any mechanical contrivance—are the three *peronei* and the *extensor communis digitorum.* In some bad cases, even this is not sufficient. As an example, take the following report.

"A patient, 15 years of age, was born with this deformity in both feet, and about two years since was operated upon and treated mechanically without relief. The patient was totally unable to walk, from the great pain occasioned when the feet were subjected to the weight of the body. Upon attempting to adduct the feet, the peroneii and common extensor were rendered extremely tense; upon attempting to flex the foot, the tendo-Achilles was also tense; and upon an attempt to depress them, the anterior tibial and extensor proprius pollicis were also tense, there being the smallest possible amount of motion in the joint, and that of an unyielding character. I therefore proposed the division of the tendons of the whole of these muscles as the only prospect of relief, which was assented to. I first divided the peroneus longus and brevis, then the extensor communis, digitorum, and peroneus tertius, afterwards the anterior tibial and extensor proprius pollicis, and lastly the tendo-Achilles. Lint and bandages were applied, and allowed to remain on a week, the patient having suffered no pain beyond that immediately following the operation. At the end of that time I applied one of Scarpa's shoes, and flexed and extended the foot alternately night and morning, so that the uniting medium of each tendon might be acted upon, the foot being adducted the whole time. At the end of three weeks the motion of the joint was restored perfectly, and the patient possessed the power of flexing and extending it at will." P. 77.

*Genu valgum,* or *knock-knee.*—This deformity is almost always of post-natal origin: Mr. Tamplin has never met with a congenital case. It may very generally be traced to debility, either hereditary, or the result of painful dentition, fevers, hooping-cough, &c. Long standing will necessarily aggravate the mal-position, if once there be any tendency to it; the weight of the body, pressing down in an oblique direction, must have the effect of approximating the knee-joints. It is a very common deformity among the poor, and is often associated with irregularities of the spine, or with rachitic curvature of the bones of the leg, and, frequently, of the femur also. Sometimes one knee only deviates from its proper direction; but in most instances both joints are affected. Occasionally the deformity in one knee is accompanied with outward inclination of the other, giving the patient a most curious appearance, in walking, of one joint running after the other. *Genu valgum* is frequently combined with spurious valgus of the ankle-joint. The internal condyle of the femur always appears considerably larger than usual; but this is only in appearance; there is no positive increase in the size of this osseous protuberance. The internal lateral ligament is always much stretched, and the crucial ligaments also are generally relaxed and elongated. Hence it comes that the articulating surfaces of the femur and tibia are much more loosely tied together than in health, so that even a certain amount of rotatory motion can be exercised between them. "As the deformity continues, you will find the biceps, flexor femoris, from being constantly somewhat shortened, offer more or less resistance, which resistance becomes proportionably increased according to the length of time it has existed, on the one hand; the state of health of the patient, on the other: so that even in children you will find this muscle, together with the fascia lata, to which the vastus externus is attached, exceedingly rigid and tense upon any attempt to place the leg forcibly in a straight line."

*Treatment.*—In weak ailing children, when the deformity is slight, we must trust chiefly to the improvement of the general health by the use of alteratives, mild chalybeates, and a nourishing diet. The use of the rocking-horse is of service; as the child, in clinging to it, naturally adducts the legs, and thus everts the knees. A straight wooden splint may be applied to the limb through its entire length, and secured with straps and bandages. If the deformity be more decided, Mr. Tamplin recommends the employment of a particular splint, made of two zinc plates; the one plate to correspond with the thigh, the other with the leg; but, instead of a screw, a straight piece of iron or wood attached by a hinge to the centre of each of the portions of the splint on the outside. The zinc, from being soft, admits of being applied close to the limb, and can be fixed by means of strapping in the position in which the joint is; a webbing strap passed round the knee and over the connecting piece of iron will, by gradually tightening it, effectually straighten the limb. In some I have also added a joint in the centre of the iron to correspond with the knee-joint, so as to allow of flexion and extension, but I prefer the knees being kept extended.

The division of the tendon of the *biceps flexor*, although it would unquestionably expedite the cure, should not be resorted to, unless the tendon be found very tense and resisting. The peroneal nerve is liable to be cut in the operation. This *malaventure* occurred in two of our author's cases. In both, paralysis of the flexors of the foot was the result; but this gradually passed away in the course of from four to ten weeks, and the patients eventually regained the entire use of their feet. After the wound of the integuments has healed, "you then apply the splint, and proceed with the extension more or less rapidly, according to the severity of the case, the rigidity met with, and the pain experienced by the patient; for I need not tell you, that the restoration of the limb to its natural position, after years of its being kept in the malposition, is always attended with pain; the pain, however, should never be allowed to interfere with the appetite or rest of the patient: this you may regard as a guide in the treatment. I

am now speaking, recollect, of the pain in the joint itself, not that which may be occasioned by undue pressure, which last ought to be immediately relieved; you must at all times be careful that the pressure is uniform throughout the entire extremity, as you may be inconvenienced, and have the treatment retarded, by a slow and open wound. After the limb is restored to its perfectly straight or natural position, you will order upright supports from the hips downwards, and keep the knee-joint in the straight position during the time exercise is taken: the leg may be flexed and extended at other times. These supports must be continued night and day, until the patient is enabled to stand and walk without the joints yielding, and must not be omitted before, as a relapse will certainly be the consequence, independently of prolonging the treatment."

In some severe and aggravated cases of knock-knee, it is occasionally necessary to divide the *vastus externus* and the *fascia* lata immediately above the external condyle of the femur, as well as the tendon of the *biceps*.

With respect to the use of irons in the treatment of knock-knee, the following practical observations of our author merit notice.

"In slight cases, in the adult or youth, irons alone, the knees being kept straight, perfect the cure of this deformity, and in a short time, compared with the more severe. * * * It is of no use to attempt to straighten the legs solely with irons, if the deformity exists to any extent, as the legs will rotate in them, and the thigh and leg become everted, which disguises, but does not relieve the deformity. Irons are only of use in severe cases, after the legs are straightened, in maintaining the position, by assisting the joints to bear the superincumbent weight of the body during the time they are incapable of doing so unaided, and allowing exercise to be taken whilst the ligaments are gaining strength, which exercise improves the general health and strength of the patients, and enables them to follow their usual occupations without risk of relapse." P. 128.

*Genu extrorsum* is invariably combined with bow-leg, or curvature outwards of the tibia and fibula. It is very common in weak rachitic children. With respect to treatment, we need not say that the general health must be diligently attended to, and that everything must be done to invigorate the constitution.

"The mechanical means we adopt consist of straight splints on the inner side of the leg, extending high up above the knee, and below the internal malleolus, well padded at the points of pressure, and webbing straps applied round the leg and splint, so that a constant steady pressure may be kept up: it is only by the most gradual and uninterrupted treatment that good can be obtained, for you must recollect that not only the knees, but the bones also are affected, and a child cannot bear any amount of continued pressure; it must be so applied that the child is subjected to no pain. This rule must invariably be your guide; you can in this way in young subjects overcome the deformity, and if the splints are carefully applied, the little patient can walk with greater comfort and firmness with them than without them. Irons have been and are daily used; I object to them, because you cannot keep up such uninterrupted support as you can with a webbing strap and splint, and without this, of course, all treatment is useless. It will occupy many months, which it is as well to inform the patients or friends, as they will become impatient and dissatisfied. With regard to those who have been thus afflicted for years, when the bones are consolidated, it is a question if you should try or advise any treatment: you must recollect the articular surfaces are not in fault; therefore, if you straighten the leg at the expense of the articular surfaces, you cannot expect the patient to be enabled to manage without a support. We have had one patient in the Charity, above twenty-six years of age, who was determined to have something done, and who submitted with the prospect of wearing irons for life; in this case I divided the semi-membranous and tendinous tendons, straightened the legs about half, by means of the splint which I adopt in knock-knee; the splint being applied on the inner side of the leg. A great improvement in his appearance is the result, but not such as would have induced me to have submitted to the treatment, or advise its being done in similar cases." P. 133.

*Contraction of the Knee in the flexed position.*—This deformity is very rarely congenital. Most frequently it is the effect of injuries or idiopathic diseases of the joint or its envelopes. At other times, it is a result of paralysis or permanent spasm of the muscles of the thigh, induced by injuries or a morbid state of some part of the cerebro-spinal axis. As the flexors are more powerful than the extensors, they predominate, and keep the joint in the bent position. But in addition to this cause, there may be more or less firm adhesions within the joint itself. If such be the case, all motion, even further flexion, of the joint is impossible, or at least very much limited; whereas, when the cause of the stiffness is external to the joint, we can generally bend it somewhat more than it usually is, although all extension may be impracticable. The circumstance too of the hamstring tendons becoming more tense, on our attempting to extend the joint, is an important diagnostic symptom: as this may be supposed to indicate that the chief resistance resides in these muscles.

Mr. Tamplin's remarks on Scrofulous disease of the knee-joint, or White Swelling, are somewhat peculiar: for he seems to contend that, even when there is destruction (ulcerative we suppose) of the cartilages and bones, amputation of the limb is very rarely called for; the diseased action being not of a truly malignant nature.

On the important subject of treatment he says:

"It requires the joint to be kept at rest, or nearly so, and a uniform support to the entire limb; the natural temperature maintained, and the general health supported, and this most uninterruptedly followed up. By these means I believe that most diseases of the joints, in young subjects, provided they are not malignant, which I do not believe they can be considered, may be cured; and even if the patients have suffered from destruction of the joints and of the bones, or that portion forming the articulating surfaces,—although all motion may be out of the question,—yet I distinctly maintain that an ankylosed knee, with the use of the foot, is far preferable to a wooden leg. The most favourable position may be selected during the period the restorative process is going on. In the majority of cases of strumous disease, the joint itself is but slightly affected, although the surrounding parts are one mass of disease. The inflammation consequent on the irritation of that disease is of a very chronic character, and does not appear in those cases to risk the integrity of the joint, although there may be, and generally is, an increased secretion of synovia, but beyond this the joint does not appear to suffer; at least, in an immense number of cases. I have witnessed the most frightful amount of disease, appearing in a superficial view to exist in the joint itself, and yet the joint remain perfectly sound, with the exception of the increased quantity of synovia." P. 150.

These statements, we fear, will not command general assent. As long, indeed, as the general health does not materially suffer from the articular disease, every means should be employed to save the limb, and none are better than those recommended by our author; but surely he would not delay having recourse to amputation, if symptoms of hectic irritation and wasting had once fairly set in, and resisted the employment of all remedial means. Certain it is however that, even under these unfavorable circumstances, a patient will sometimes recover with an anchylosed joint; but this is not common. The following case was one of those happy exceptions: the treatment reflects much credit on Mr. Tamplin's skill and perseverance:—

"The girl, about 8 years of age, was sitting on the bed in the most miserably emaciated condition, and was stated to have been suffering from disease in the knee between two and three years. There were five openings, two on the outside, just above condyles, one on the inside, and two on the patella. The three first communicated with the femur, the two last with the patella. The parents stated that at least half a pint of matter was discharged daily. Of course, this was somewhat exaggerated, but an immense discharge was then evident, of that peculiar, thin, unhealthy secretion, common in scrofulous diseases. The knee was contracted beyond a right angle, so that you could not pass a thin piece of sponge between the leg and thigh, in the situation of the popliteal space. The knee itself was swollen, and distended with fluid. She was suffering from acute hectic fever, with profuse perspiration and severe pain on the slightest motion. The pulse was fluttering, the bowels relaxed; in fact, the child presented the appearance of a person in the last stage of consumption. I ordered her an opiate, with the hyd. c̄. cretâ, every night, the conf. aromat. and ext. cinch. three or four times a day, a soft linseed-meal poultice, made by stirring the meal into boiling water, over the whole of the knee-joint, covered with oiled silk. A tin splint, bent at the angle at which the knee was flexed, with pads, and retained by means of a flannel bandage, from the toes upwards above the knee, thereby keeping up the natural temperature of the entire limb, as well as a uniform gentle support; together with a nourishing diet, consisting of eggs, milk, meat, beer, and then two glasses of port wine daily, commencing the stimulants by degrees. In a few days the general irritability subsided, and the pain in the knee was relieved, the hectic left her, and the discharge altered its character. In fourteen days she could be moved without a complaint.

"This treatment was continued six months, varying the tonic, at the end of which time all the openings were closed, and the swelling of the joint almost gone. The leg was extended in the most insensible manner, by gradually straightening the splint during the period the disease was subsiding, and without pain being produced. As soon as the openings were healed, I supported the joint with emp. cerat. saponis, and continued the bandage and splint, and in twelve months the leg was brought into the straight position, and the girl could use it without any assistance. I then directed the joint to be exercised daily, by forcibly flexing and extending it, as far as the feelings of the child would admit, and in this way the motion of the joint was restored, the muscles of the thigh and calf developed themselves, and a perfectly useful limb is the result. In this case amputation was advised by several surgeons as the only means of saving the child's life." P. 152.

The best way of effecting gradual extension of a flexed knee, when this is deemed advisable, is by means of a double splint for the thigh and leg, having a hinge in the ham, and provided with a male and female screw beneath. If the deformity does not yield, then we must have recourse to section of the tendons of the inner and outer hamstring muscles, and of any dense bands of fascia that may be felt. After the knee has become extended, it may be necessary to divide the tendo-Achillis, to relieve the motions of the foot.

*Contraction of the Knee-joint in the extended position* is not a common affection, either congenital or post-natal. In one congenital case, Mr. Tamplin divided the *rectus* muscle, about an inch and a half above the patella, with decided benefit:—" the result has been most satisfactory." It will always be right to try the effects of gradual mechanical flexion by means of a male and female screw attached to the double splint, in cases occurring in consequence of inflammation or any other affection of the joint, provided always the flexion can be effected without great force. We may fairly presume that our author condemns the recent practice of some French surgeons on this point, when we find him expressly saying that " in cases which have arisen from adhesions in the joint, when the joint is immoveable, and when upon forcibly attempting to flex it no pain is experienced, I would not advise your having recourse to any means of restoration, as the patient possesses a limb that is comparatively useful, although limited in its motions; whereas, if you rupture the adhesions, the probability is you will not eventually improve the condition of the patient—at least, I have not seen any permanent good arise from such treatment: although you may succeed in flexing the joint for a time, yet the chances are that it will return to its former state, as it can scarcely be expected that the secretory surface of the synovial membrane, or that the ruptured extremities, will assume a condition compatible with free motion."

*Contraction of the Hip-joint* is generally the result of cerebral or spinal irritation, injury to the spine, position, rheumatic or idiopathic arthritis, or of strumous disease in the joint or its neighbourhood. The limb is often adducted, so as to be drawn over the other one, at the same time that it is fixedly bent; and not unfrequently the feet also are deformed. In incipient cases, Mr. T. recommends that a trial should be made of an instrument which he has contrived, and " which, by its fixing the pelvis, and being also attached by the broad webbing strap to the chest and abdomen, will enable you to keep up any amount of extension on the thigh, first bandaging the foot, leg, thigh, and hip, with a flannel roller; for, as I mentioned to you when speaking of scrofulous disease of the knee-joint, unless the natural temperature is kept up, the restorative process cannot go on.

" In cases occurring in the poorer classes, whose means do not allow of their procuring an instrument, I have had recourse to a straight board, corresponding with the size of the back, with an extended portion to correspond with the thigh contracted, webbing straps being nailed on each side of the board, so that the abdomen, chest, and pelvis, may be secured tolerably well: of course a pad must be used, to prevent any undue pressure being made. With this I have succeeded in bringing down the thigh, and preventing a contraction from taking place. You cannot, of course, restore any amount of motion, and your attention must therefore be directed to bringing the thigh straight with the pelvis, which enables a patient to use the leg with comparative ease, and without the use of crutches. In young subjects, provided the disease has subsided, and a short time only has elapsed, the contraction may, as a rule, be removed without an operation, but if in the course of extension any of the superficial muscles are found to be rigidly tense, a division of them had better be effected."

In one case, where the thigh was permanently flexed at a right angle—the result of a disease in the joint—Mr. T. divided the *rectus, adductor longus,* and *tensor vaginæ*; and, after the healing of the punctures, he kept up a steady and gradually-increased extension of the limb. The result was partially successful; for the limb was brought into a semi-bent position, so that the boy could get about tolerably well with the aid of a stick, whereas, before, he had been always obliged to use crutches. Allusion is also made to another patient, æt. 14, in whom the *adductor longus* of each thigh, the flexors of both knees, and the tendo-Achilles in each foot were divided, and who "is now walking about with comparative ease." The surgeon, therefore, is not to be discouraged even under very unpromising circumstances, provided he has reason to believe that the joint itself is not seriously altered.

The deformities hitherto described have been those of the lower extremities. The 11th, 12th and 13th Lectures are devoted to the description of Curvatures and Distortions of the Spine; but, as this subject engaged our attention in the last number of the Review, we need not resume it at present. All that we shall now do is to make a few observations on one or two practical points.

The general character of Mr. Tamplin's therapeutic directions leads us to suspect that he is much more friendly to the continued use of mechanical supports, in the treatment of spinal deformities, than most enlightened surgeons in the present day are in the habit of recommending. For example, in the treatment of confirmed posterior curvature, when the vertebræ are found upon examination to be rigidly fixed, he advises that "an instrument,"—consisting of a band round the pelvis, two side crutches for the shoulders, a back-board for the entire length of the spine, and a head-support—"should be worn night and day; as, unless the extension is constantly maintained, little if any good can by any possibility be effected;" it is added, "a continued pressure can be kept up upon the prominent portion of the curve by the backboard attached, and the shoulders held back by straps."

We pity the poor patient, who is doomed to sleep thus "cribbed, cabined and confined." Nor can we believe that such treatment is ever necessary, except in some extreme cases of caries of the vertebræ.

In slight cases of lateral curvature, Mr. T. recommends the use of Tavernier's belt. "This instrument, if properly applied, and carefully kept in its position, will cure any slight case of curvature by the constant support and pressure that it is capable of effecting on the projecting ribs. It must, however, be worn night and day, so that no return of the spine to the mal-position is admitted of; as here, as well as in the deformities I have previously directed your attention to, unless the position is constantly maintained, the ligaments cannot recontract, neither can any resistance which those on the concavity of the curve offer be overcome: this, therefore, is indispensably necessary."

This advice is altogether most injudicious. Tavernier's belt, although less injurious than many other instruments, is far from being unobjectionable. The constant pressure, that is kept up upon the muscles of the projecting side, is itself no small disadvantage. It is obvious too, that, if there be any tendency to a second curvature in the opposite direction lower down in the spine, the continual use of the belt in question may rather aggravate than lessen the evil. The only manual exercise, we may remark, which our author approves of, is the use of a common pulley, fixed in a convenient position, for the arm corresponding with the concave side of the spinal curvature.

"I most decidedly object," says he, "to the exercise of the muscles on both sides of the spinal column, during the time the curve is in existence, as is most commonly done in a variety of amusing and not less expensive ways to the patient, as by so doing a direct obstacle is maintained, and kept in active operation to prevent the removal of the curvature—a practice so opposed to common sense that one is at a loss to assign a reason for it, and a practice entirely opposed to the course adopted, and found to be of such essential importance, in the treatment of other deformities without exception." P. 236.

Our author is too sensible a man to give his assent to the preposterous practice of dividing any of the dorsal muscles in the treatment of lateral curvature of the spine; the honour of this invention is purely French.

The last Lecture treats of Wry-neck, and Contractions of the lower jaw, and of the arm and hand. Our notice of these deformities must be very brief. The usual causes of *Wry-neck* are rheumatic inflammation of the sterno-mastoid, paralysis of the same muscle, and disease of the cervical vertebræ. The paralytic affection of the muscle is apt to be produced by dentition, worms, or any of the numerous causes which interfere with the health of a child. The deformity is very rarely congenital. The muscular contraction is limited sometimes to the sternal, at other times to the clavicular, portion of the sterno-mastoid. When it is deemed adviseable to have recourse to section of the muscle, the operation must be performed with great care and delicacy, in order to avoid any of the important vessels of the neck.

The following case of rigid *Contraction of the Lower Jaw* is interesting: its successful treatment reflects the highest credit on Mr. Tamplin's skill.

"Sometime since I was consulted by a patient, aged 11 years, whose mouth was, and had been for two years rigidly closed. Upon introducing my finger in the angle of the mouth, a dense indurated cicatrix was found to exist on either side, which effectually prevented the patient from opening his mouth in the slightest degree. His mother stated that for the period mentioned he had lived by suction only. Many surgeons had been consulted, but all had given an unfavourable opinion. I determined to divide the cicatrices by a puncture externally, which was done in the following manner. The patient was laid on a table, the head being supported with a pillow, an assistant holding the head. I introduced a small scalpel perpendicularly upon the middle of the masseter muscle; then withdrew it, and inserted a long narrow blunt-pointed knife, carrying it down to the bone, depressing the handle so that I could pass it horizontally forwards, until I felt the point at the anterior edge of the cicatrix, to the outer side of the mucous membrane, which I was enabled to do by keeping the finger of my left hand within the angle of the mouth. I then turned the sharp edge towards it, and gradually divided the band transversely. By this proceeding the mucous membrane of the mouth was left uninjured, the cicatrix alone being divided; so that there was no possibility of hæmorrhage. Pledgets of lint were applied over the point of puncture, as well as over the line of incision, and secured with strapping and bandage. Scarcely any pain followed the operation; and, although only one cicatrix was divided, the boy could open his mouth to the extent of about the eighth of an inch.

"Four days afterwards the puncture was healed, when I divided the cicatrix on the opposite side in a similar manner, and with a similarly satisfactory result. At the end of four days following the last operation, I applied an instrument made to fit the teeth of the upper jaw, which acted as a fulcrum, and with small narrow blunt steel hooks, which I introduced over the teeth of the lower jaw, and afterwards attached to the instrument by means of a screw, with which I gradually opened the mouth. During the night the instrument was removed, and small wedges of ivory were introduced, to which were attached pieces of tape, so that there should be no possibility of their getting into the mouth or throat. At the end of fourteen days, the boy was able to eat any kind of solid food, and, from his mother's account, was eating the whole of the day. He rapidly improved in health and strength, and, with a continuance of the use of the instrument, the cicatrices became gradually absorbed." P. 250.

The remainder of the last lecture is occupied with a description of the contractions of the shoulder, elbow, and wrist-joints, and also of the fingers and toes. It is not necessary to detail particulars; and here we must close the present article.

On the whole, this work of Mr. Tamplin's may be consulted with much profit by the surgical reader, and we therefore strongly recommend our professional friends generally, and especially such as reside in the country or are at a distance from an orthopædic institution, to have a copy of it beside them. In the event of a second edition, the author will do well to make more frequent reference to other writers upon the subjects discussed, and to compare his own opinions and practice with their's. This is but fair to them as well as to himself.

